# Study of the Microstructure Characteristics of Three Different Fine-grained Tailings Sand Samples during Penetration

**DOI:** 10.3390/ma13071585

**Published:** 2020-03-30

**Authors:** Yueqi Shi, Changhong Li, Dayu Long

**Affiliations:** Key Laboratory of Ministry of Education for High-Efficient Mining and Safety of Metal Mines, University of Science and Technology Beijing (USTB), Beijing 100083, China; lch@ustb.edu.cn (C.L.); ldayu2018@163.com (D.L.)

**Keywords:** X-ray microtomography, microstructure characteristics, infiltration damage

## Abstract

This paper explores the microstructural evolution characteristics of tailings sand samples of different types of infiltration failure during the infiltration failure process. The homemade small infiltration deformation instrument is used to test the infiltration failure characteristics of the tailings sand during the infiltration failure process. Evolutionary characteristics of the internal microstructure pores and particle distribution were also studied. Using CT (computerized tomography) technology to establish digital image information, the distribution of the microscopic characteristics of the particle distribution and pore structure after tailing sand infiltration were studied. Microscopic analysis was also performed to analyze the microscopic process of infiltration and destruction, as well as to see the microscopic structural characteristics of the infiltration and destruction of the total tailings. The test results show that there are obvious differences in the microstructure characterization of fluid soil and piping-type infiltration failures. Microstructure parameters have a certain functional relationship with macrofactors. Combining the relationship between macrophysical and mechanical parameters and microstructural parameters, new ideas for future research and the prevention of tailings sand infiltration and failure mechanisms is provided.

## 1. Introduction

Tailings sand is a solid waste material with a low content of useful components that remains after crushing and sorting the ore. This material is not suitable for further sorting under current economic and technological conditions. As a raw material for building tailings dams, it is a man-made three-phase dispersion medium with special structural characteristics as well as physical and mechanical properties [1,2]. The particle size of fine-grained tailings is very small, and thus, its specific surface area is large; its reactivity is also high [3,4]. Therefore, compared with ordinary tailings, the enrichment of fine-grained tailings can easily lead to lens bodies or a weak tailings dam interlayer. Furthermore, the strength of tailings sand, as sandy soil, is not high. When infiltration damage occurs, tailings sand will leak intensively in weak places, such as the dam foundation and abutment, forming voids inside the dam body, and even causing the local collapse of the dam body (tailings dam break). Scholars worldwide have carried out numerous experimental studies on the percolation and failure of natural soils and have made some progress in the study of the mechanical properties of tailings. However, most of them have discussed the macromechanical properties of tailings and the deformation and evolution of the fine content and microstructure. Studies on the characteristics and mechanical behavior of tailings are still scarce [5,6,7,8,9,10,11,12,13]. At the same time, high-resolution X-ray microtomography (micro-CT) has been widely used as a nondestructive technique that can perform three-dimensional (3D) imaging to analyze the internal characteristics of objects [14]. Although this technology has been widely used to investigate the microstructures of rocks and soils, it has not been applied to tailings sand.

This paper mainly studies the characteristics of the microstructural changes of different infiltration and destruction types in the tailings sand seepage process and provides new ideas for the study of tailings sand infiltration, failure mechanisms, and prevention measures. Taking the fine-grained tailings in Makeng, Fujian, as the research object, a homemade small osmotic deformation instrument was used to test the osmotic failure characteristics of tailings sand samples with fine contents of 30%, 50%, and 70%. The evolution of the internal microscopic pores and the particle distribution of fine-grained tailings sand during osmotic failure were characterized. During the process of osmotic failure, the head was stepwise loaded with four heads of water until the sample was damaged, and X-ray microtomography (micro-CT) was used to scan the tailings sand samples under the four head pressures. Using the VGStudio max 3.0 and Avizo 9.0.1 visualization software provided by Sanying Precision, Tianjin, China, the three-dimensional (3D) reconstruction of the scanned sample data was performed, the digital image information was established, and the microscopic characteristics of the particle distribution and pore structure of the tailings sand after infiltration and destruction were analyzed. During the infiltration and destruction process of granular tailings, the characteristics of the internal microstructure voids, and the changes in the particles are summarized. The characteristics of the infiltration and destruction of fine-grained tailings are summarized.

## 2. Materials and Methods

### 2.1. Test Tailings Sand Material

**Figure 1:**
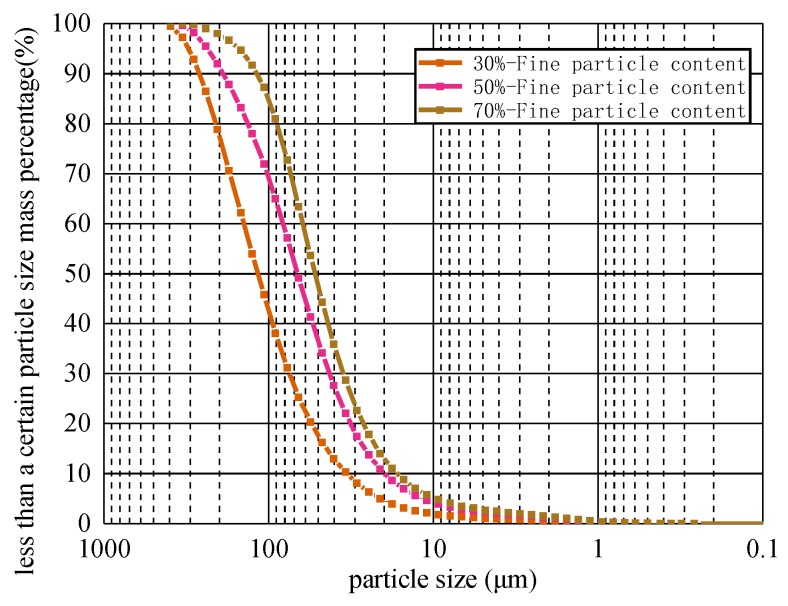
Gradation curve of tailings sand.

All of the tailings sand materials used in the tests were sampled from Fujian’s Makeng iron tailings pond. The gradation curve is shown in Figure 1. Figure 1 shows that the fine grain content, with grain sizes less than 0.075 mm, is between 15% and 50%, and this material is referred to as silty sand (SM). At the same time, the coefficient of nonuniformity (Cu) of the tailings sand material is greater than 5; that is, the sizes of the coarse and fine particles in the tailings sand are very different, and the fine particles can easily fill in the pores formed by the coarse particles to form a better skeleton structure.

### 2.2. Test Device and Scheme

#### 2.2.1. Small Osmotic Deformation Instrument

**Figure 2:**
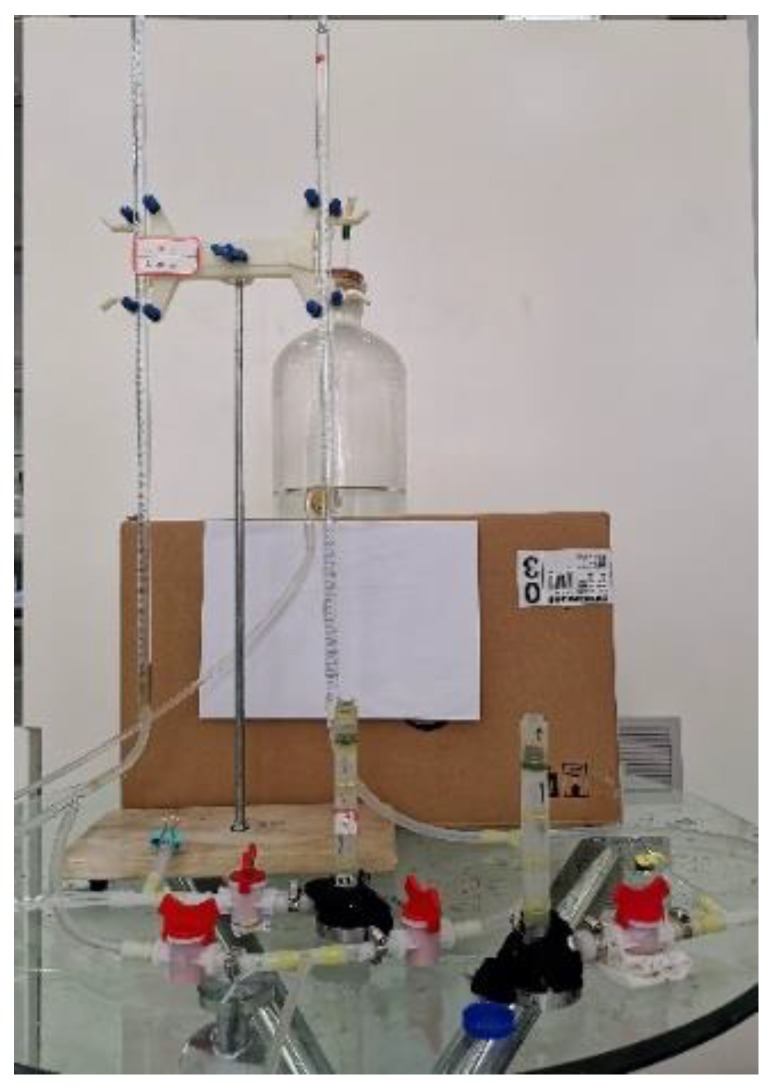
Small osmotic deformation instrument.

The test device used is a set of homemade small osmotic deformation instruments improved by an ordinary osmometer, as shown in Figure 2. The instrument consists of a test container, a pressurization device, a water supply device, and other parts. The test container is a transparent acrylic round tube with a height of 50 mm, an inner diameter of 10 mm, and an outer diameter of 14 mm. Furthermore, the device is a testing and filling device for tailings sand so that test phenomena can be conveniently observed. Since the particle size of the tailings sand used in this test is relatively small, and its permeability coefficient is small, the pressure device is a millimeter-scaled variable head pipe. The water supply device is a transparent water supply bottle.

#### 2.2.2. X-ray Micro-CT

**Figure 3:**
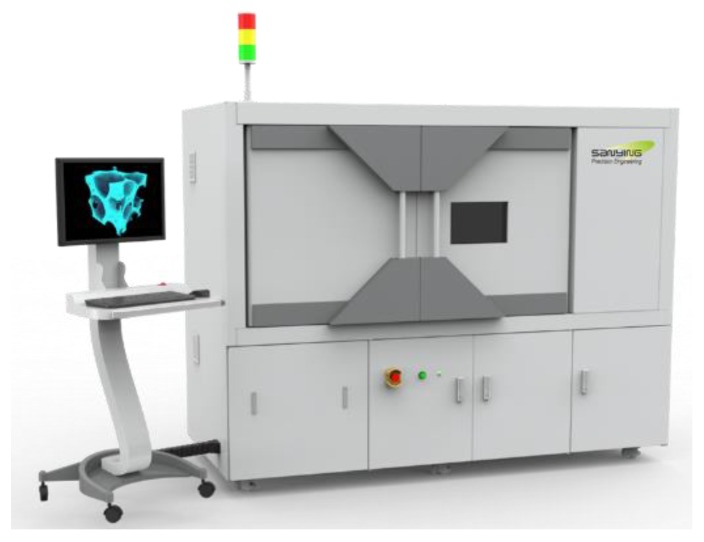
nanoVoxel-3000 micro-CT.

The instrument used for tailings sand sample testing is a nano Voxel-3000 micro-CT provided by Sanying Precision, Tianjin, China, as shown in Figure 3. The instrument has advanced nondestructive 3D imaging and image analysis capabilities, with secondary optical magnification and a spatial resolution of up to 0.5 μm. Using this equipment, the 3D visualization of the internal microstructure of materials can be nondestructively investigated.

In this test, the test voltage of the micro-CT equipment is 140 kV, the test current is 70 μA, the exposure time is 0.42 s, the standoff distance (SoD) from the source to the sample is 13.6 mm, and the distance from the source to the flat panel detector (SDD) is 279.6 mm. The resolution is 6.16 μm, which can distinguish the characteristics of pores with pore sizes greater than 6.16 μm and tailings sand particles. This experiment adopts the continuous scanning method of a cone-beam with a scanning rate of 0.25°/frame and a total of 1440 projections. Numerous layers (1536) of the sample are cut longitudinally, and each layer thickness is 6.16 μm. Finally, 1536 two-dimensional (2D) slice images of 1800 × 1800 pixels are obtained. The parameters of the CT scan of the tailings sample are shown in Table 1.

#### 2.2.3. Test Method and Process

To obtain the process of tailings sand infiltration and failure, the test was simulated by gradually increasing the head difference.

**Figure 4:**
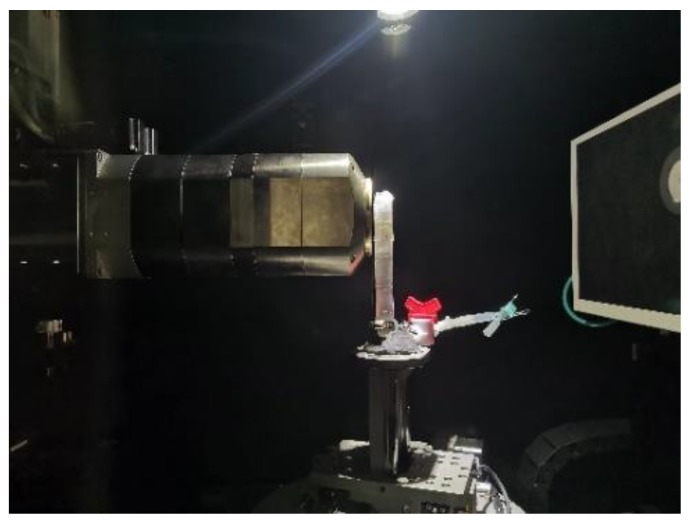
CT scan of the tailings sand sample.

According to the critical head penetration failure heads of three different fine-grained tailings sand samples, the heads under test loading are divided into four grades. The top three grades are as follows: level 2 is the head value without seepage damage, level 3 is the critical head damage head value of the sample, and level 4 is the head value after seepage failure. After each stage is loaded stably, a CT scan is performed on the sample pressure relief, as shown in Figure 4. The meso-infiltration characterization inside the sample is obtained. After scanning, the head pressure of the seepage is increased to the next head value. According to the recorded experimental phenomena, the distribution of the head value for each stage of the sample is shown in Table 2.

During the test, attention is paid to whether the test surface has water turbidity, bubbling, or fine particle bounce, whether there is a bulge or a rise on the sample surface, and whether there are faults in the sample [15]. When the average velocity of seepage suddenly increases and is unstable or when concentrated seepage occurs on the pipe wall, the test is stopped.

During the test, the values of the seepage velocity and permeability coefficient are recorded as the head difference gradually increases (see Table 3).

When the 30% fine-grained sample is loaded with a third-level head, a slight floating soil phenomenon appears on the top of the sample. When a fourth-level head is loaded, seepage failure occurs. The seepage at the top of the sample is very turbid, and the soil is completely jacked up. This phenomenon is a piping-type infiltration failure. When the 50% fine-grained sample is loaded with a third-level head, a slight floating soil phenomenon appears on the top of the sample, and microcracks appear in the middle and lower parts of the sample. When a fourth-level head is loaded, complete infiltration failure occurs. The seepage flow at the top of the sample is very turbid, the flow velocity increases sharply, and the lower cracks further expand. Fine particle bounces appear at the top of the sample, which suggests that fluid-type infiltration failure occurs. When the 70% fine particle sample is loaded with a second-level head, the flow rate increases sharply. The surface of the tailings sample exhibits a slight support phenomenon, and there are visible cracks in the lower part of the sample. When the third-level head is loaded, complete percolation failure occurs. The seepage at the top of the sample becomes very turbid, the flow velocity increases sharply, and the lower cracks further expand. Fine-grained beating appeared on the top of the sample, and it was judged that a fluid-type infiltration failure occurred.

## 3. Test Results and Analysis

### 3.1. Three-Dimensional Reconstruction of the CT Scan of Fine-Grained Tailings Sand

**Figure 5:**
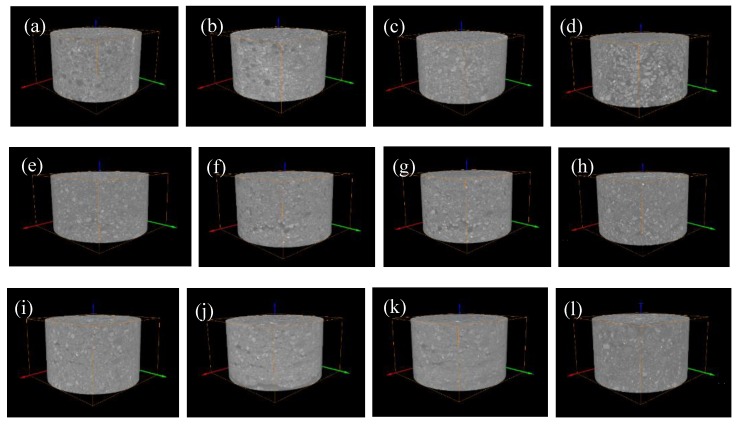
Three-dimensional reconstruction of the three fine-grained tailings samples. (**a**) First-level head of 30% fine content; (**b**) Second-level head of 30% fine content; (**c**) Third-level head of 30% fine content; (**d**) Fourth-level head of 30% fine content; (**e**) First-level head of 50% fine content; (**f**) Second-level head of 50% fine content; (**g**) Third-level head of 50% fine content; (**h**) Fourth-level head of 50% fine content; (**i**) First-level head of 70% fine content; (**j**) Second-level head of 70% fine content; (**k**) Third-level head of 70% fine content; (**l**) Fourth-level head of 70% fine content.

Cone shadows are generated in the upper and lower slices of the data generated by the CT scan, which affects the subsequent 3D reconstruction and quantitative characterization of the pore structure. Therefore, the CT layers need to be cropped in the VGStudio max 3.0 visualization software provided by Sanying Precision, Tianjin, China to obtain 1000 layers of 1800 × 1800 pixels. The 2D slice map was imported into the Avizo software for the 3D reconstruction for further analysis. The 2D slice image of the tailings sand sample obtained by cone-beam scanning is affected by various types of system noise and artifacts. The gray image obtained by the local mean filter needs to be filtered and denoised [16]. Then, the artifacts are effectively removed by the Avizo software provided by Sanying Precision, Tianjin, China, and the scanned data are reconstructed by the software’s iterative reconstruction algorithm to obtain a 3D map of the tailings sand sample under the head pressures of 1–4, as shown in Figure 5. Afterward, the image segmentation processing is performed according to the commonly used Otsu algorithm [17], and the grayscale image is binarized. Finally, the 3D pore structure and tailings sand particle structure in the data volume are extracted by the Avizo software, and a 3D pore and tailings sand particle network model of the pores and tailings sand particle spatial arrangement is generated from the micro-CT image.

### 3.2. Characterization of the Microstructure of the Pores during Infiltration Failure of Tailings Sand

**Figure 6:**
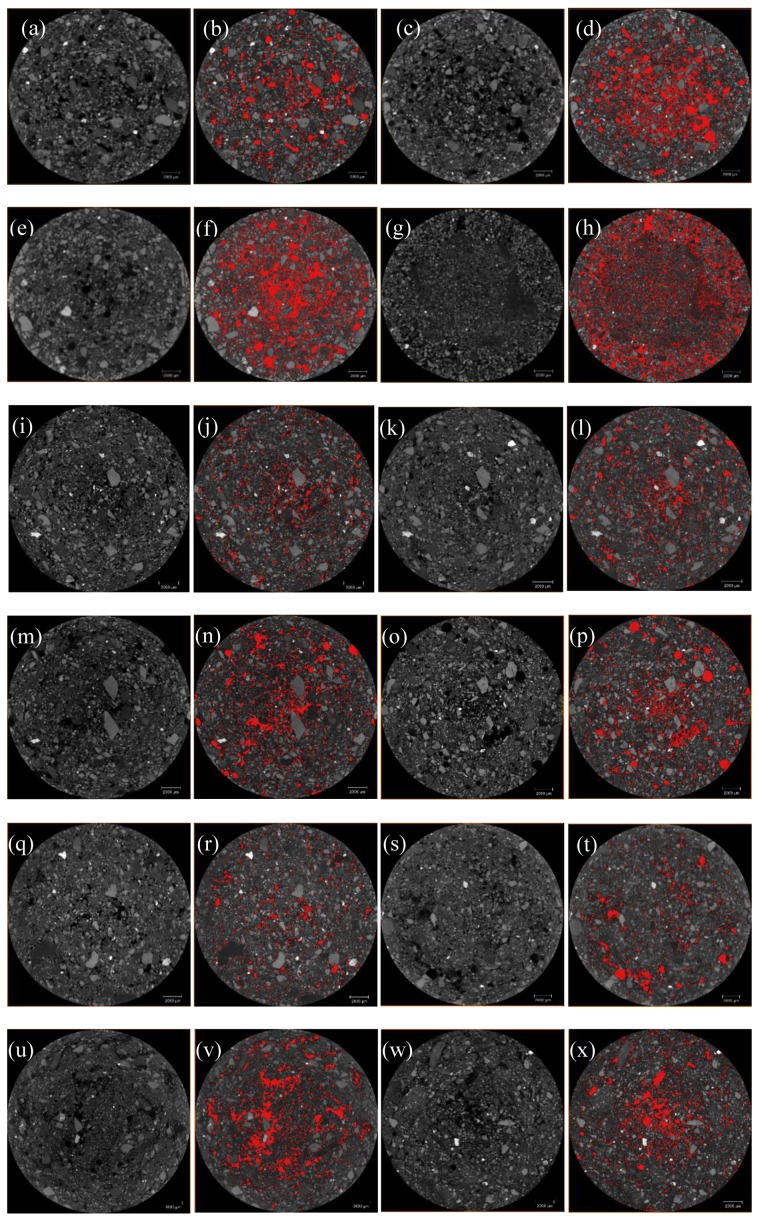
Pore threshold segmentation of the three fine-grained tailings sands. (**a**,**b**) First-level head of 30% fine content; (**c**,**d**) Second-level head of 30% fine content; (**e**,**f**) Third-level head of 30% fine content; (**g**,**h**) Fourth-level head of 30% fine content; (**i**,**j**) First-level head of 50% fine content; (**k**,**l**) Second-level head of 50% fine content; (**m**,**n**) Third-level head of 50% fine content; (**o**,**p**) Fourth-level head of 50% fine content; (**q**,**r**) First-level head of 70% fine content; (**s**,**t**) Second-level head of 70% fine content; (**u**,**v**) Third-level head of 70% fine content; (**w**,**x**) Fourth-level head of 70% fine content.

**Figure 7:**
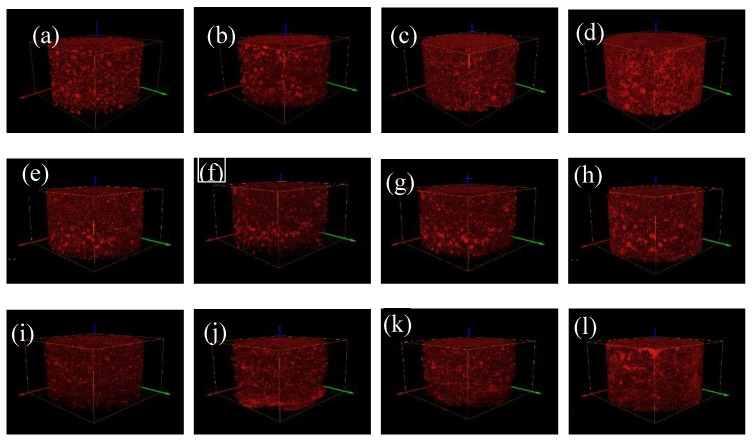
The overall pore structure of the three fine-grained tailings sand samples. (**a**) First-level head of 30% fine content; (**b**) Second-level head of 30% fine content; (**c**) Third-level head of 30% fine content; (**d**) Fourth-level head of 30% fine content; (**e**) First-level head of 50% fine content; (**f**) Second-level head of 50% fine content; (**g**) Third-level head of 50% fine content; (**h**) Fourth-level head of 50% fine content; (**i**) First-level head of 70% fine content; (**j**) Second-level head of 70% fine content; (**k**) Third-level head of 70% fine content; (**l**) Fourth-level head of 70% fine content.

Based on the previous digital image processing, to obtain the pore structure model of the tailings sand samples, the grayscale image is binarized using image threshold segmentation, as shown in Figure 6a–x. The volume rendering module of the Avizo 3D visualization software is used to directly perform the 3D reconstruction of the pore structure of the sample, and the overall 3D pore microstructure of the three fine-grained tailings sand samples is obtained, as shown in Figure 7.

It can be seen from Figure 7 that the overall pore structures of the three fine-grained tailings sand samples are fully developed, the pores are unevenly distributed throughout the sample, and the pore shape changes are diverse at the surface of the pore structure. Small pores gradually penetrate into large pores, and the pore structure under the head pressure of level 4 is the most obvious.

#### 3.2.1. Characterization of the Pore Number and Volume

Based on the 3D model of the tailings sand pore data obtained by Avizo 3D visualization software, the label analysis command in Avizo software is used to statistically analyze the sizes of the pores in the tailings sand sample, and the target pore size and number are filtered by the analysis filter command.

**Figure 8:**
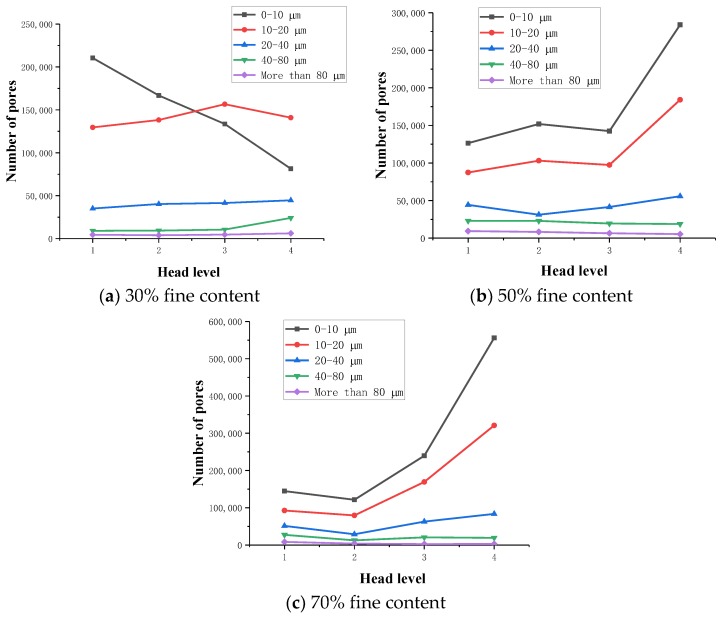
Variation in the pore number. (**a**) 30% fine content; (**b**) 50% fine content; (**c**) 70% fine content.

**Figure 9:**
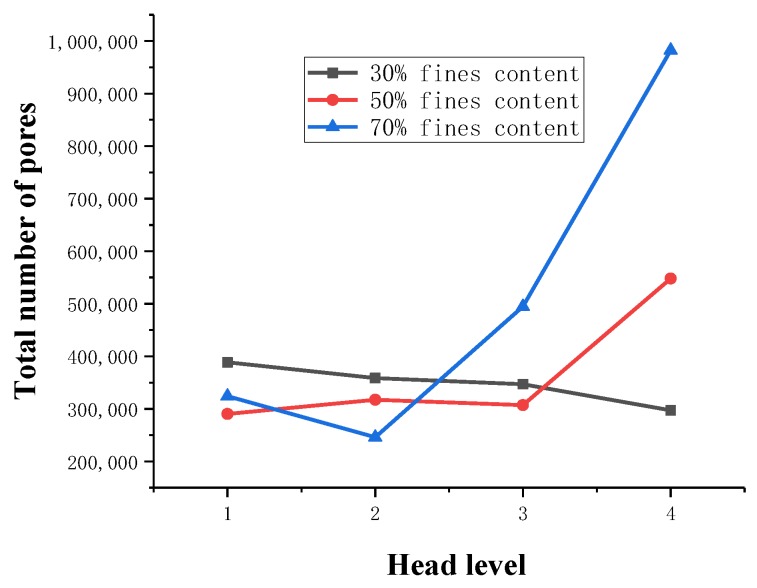
Change in the total pore quantity.

**Figure 10:**
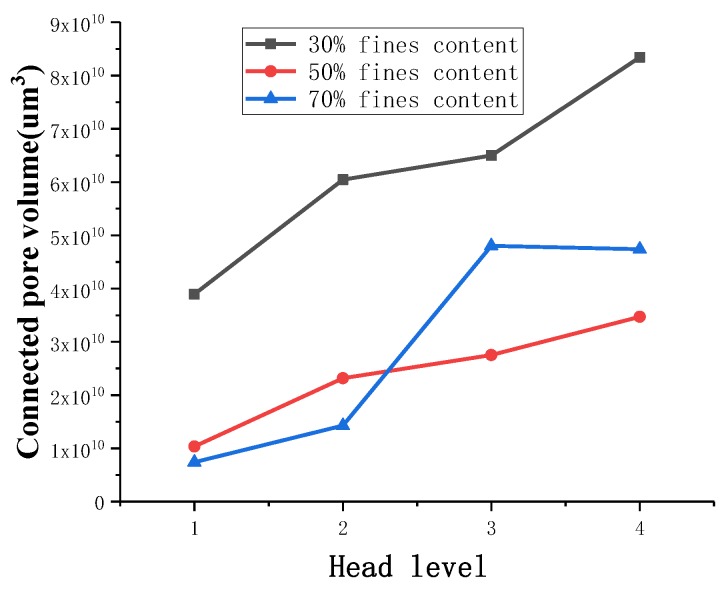
Total connected pore volume change.

Based on the actual resolution of the tailings sand sample scanning, the pore size of the tailings sand sample is divided into five categories for statistical analysis: D ≥ 80 μm, 40 μm ≤ D < 80 μm, 20 μm ≤ D < 40 μm, 10 μm ≤ D < 20 μm, and 0 μm ≤ D < 10 μm. The statistical results are shown in Table 4, Table 5 and Table 6 and Figure 8, Figure 9 and Figure 10.

As seen from the above chart:

(1) For samples with a 30% fine content, pores ranging from 0–10 µm decrease significantly during the seepage process, while pores larger than 10 µm show a relatively increasing trend, indicating that as fine particles flow and migrate, small disconnected pores are formed prior to the seepage. When larger pores are formed, the connected pores are developed, the permeability of the sample is enhanced, and the particle migration capacity is improved.
(2) For 50% fine particles, pores ranging from 0–20 µm increase during the seepage process, and pores larger than 20 µm increase during the seepage process, indicating that during this process, the fine particles migrate into the large pores due to seepage forces. In this case, the large pores are divided into several small pores, which reduce the development speed of the connected pores, thus leading to a decrease in the percolation channels inside the sample. As the percolation pressure increases, fine particles clearly flow and migrate. At this time, the number of 0–20 µm pores shows a downward trend, whereas the 20–40 µm medium pores increase. As the total number of pores increases, the number of large pores greater than 40 µm is significantly reduced, resulting in greater interaction between soil particles and fluid damage to the sample as a whole.
(3) During the seepage process, the sample with a 70% fine content has more fine content, and the connected pores are not developed. All pores are reduced during the early stage of seepage, and the force between soil particles is significantly increased. Fluid soil damage rapidly occurs, and the number of pores rapidly increases after the damage.

Through a comparative analysis, we have determined the following:

(1) The total number of pores in the 30% fine-grained sample shows a decreasing trend during the seepage process. The small pores are connected to become large pores, which enhances the sample’s permeability, and connected seepage channels are formed inside the sample. The connected pores are fully developed.
(2) In the 50% fine particle sample, the total number of pores first increases and then decreases during the seepage process. After the damage occurs, the number of pores rapidly increases. At the beginning of the seepage, the fine particle migration divided the large and medium pores into smaller pores, resulting in numerous pores. During the later stage of percolation, with increasing permeation force, the connected pores develop to a certain extent inside the sample, forming a certain percolation channel, and the particles begin to migrate with the permeation force, resulting in a rapid decrease in the number of micropores with sizes ranging from 0–20 µm. The number of medium pores of 20–40 µm increases, the number of large pores larger than 40 µm decreases, the total number of pores decreases, and the connected pores develop slowly.
(3) During the seepage process, the total number of pores in a 70% fine-grained sample is relatively small, the soil particles and the seepage force increase rapidly, and the sample quickly undergoes fluid soil failure. After breaking the ring, the number of pores increases rapidly. The connected pore volume also increases rapidly.

#### 3.2.2. Evolution Characteristics of the Connected Pores

**Figure 11:**
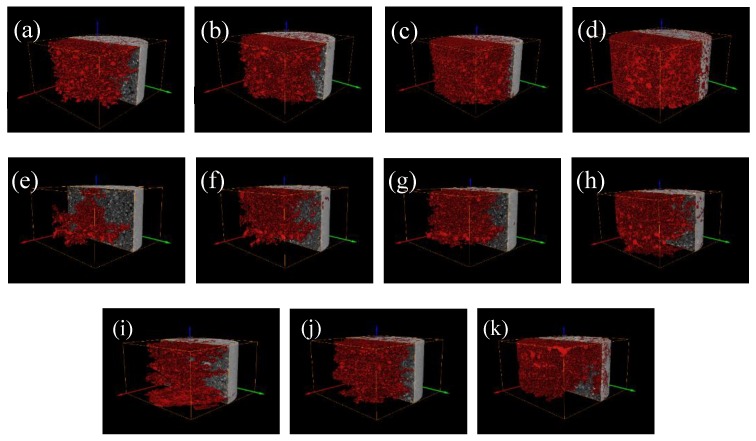
Connected pore structure under three levels of the water content at the three levels of fine particle content. (**a**) First-level head of 30% fine content; (**b**) Second-level head of 30% fine content; (**c**) Third-level head of 30% fine content; (**d**) Fourth-level head of 30% fine content; (**e**) First-level head of 50% fine content; (**f**) Second-level head of 50% fine content; (**g**) Third-level head of 50% fine content; (**h**) Fourth-level head of 50% fine content; (**i**) Second-level head of 70% fine content; (**j**) Third-level head of 70% fine content; (**k**) Fourth-level head of 70% fine content.

On the basis of obtaining the overall pore structure of three fine-grained tailings sand samples, the connected pore structure of the samples with 1–4 head pressures along the seepage direction is extracted by the Avizo 3D visualization software (see Figure 11). Then, the volume of the connected pores is counted.

With increasing head pressure, the connected pore volume of the 30% fine particle sample gradually increases. Fine particles migrate and flow under the action of the seepage, small pores easily penetrate into large pores, and the increase is obvious. The connected pore structure of the 50% fine-grained tailings sand sample runs through the entire tailings sand sample along the seepage direction because this sand sample is finer than the 30% fine-grained tailings sand sample. Increasing the particle content reduces the pores between the particles, and thus, the connected pores passing through the entire sample are relatively insignificant at the first head pressure. As the head pressure gradually increases, the connected pores increasingly develop around them. This behavior indicates that as the seepage force increases, the fine particles migrate and flow to expand the small pores into larger pores, increasing the connectivity of the sample. As the connectivity increases, the damage to the sample is more serious, and the development of the connected pores is more obvious.

The 70% fine-grained tailings sand sample did not pass through the entire sample through the pores under the first-level head. As the head pressure is increased, the fine particles migrate and flow. At this time, the connected pores penetrate the entire sample. The 70% fine-grained tailings sand sample has a smaller proportion of pores than the 30% and 50% fine-grained tailings sand samples due to the large proportion of fine content, and the connectivity of the pores is poor. From level 2 to level 4 heads, with increasing seepage force, the connected pores develop from the sample center to the surrounding area. As the head pressure increases, the connected pore volume tends to increase first and then decrease. The 70% fine content tailings sand sample has a large proportion of fine content, and the pores between the particles are small. When the sample begins to infiltrate and break, the volume of the connected pores does not increase significantly. From the 2^nd^ to the 3^rd^ head, due to the continuous increase in the seepage force, a large number of fine particles migrate and flow, which makes the volume of the connected pores increase sharply to 4.80 × 10^10^ μm^3^. From grade 3 to grade 4 heads, the volume of the connected pores slowly decreases, indicating that the tailings sand sample under the grade 4 head pressure is severely damaged. When a CT scan is carried out when the head pressure is unloaded, a large number of fine particles are uniform due to settlement.

#### 3.2.3. Change Characteristics of the Porosity Layer by Layer

**Figure 12:**
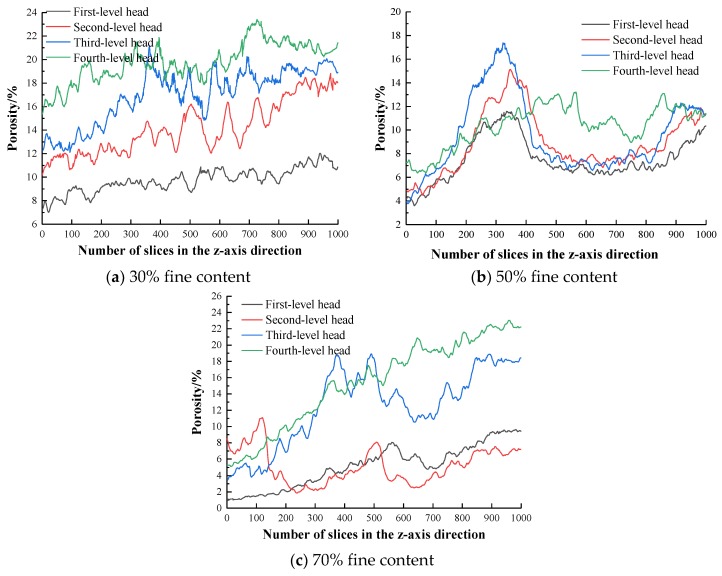
Layer-by-layer porosity statistics of the three fine-grained tailings sand samples.

Based on the Avizo 3D visualization software, the pores are extracted after the threshold segmentation of three kinds of fine-grained tailings sand samples, and statistical analyses of the layer-by-layer porosity of the 1000-layer 2D slice map of three kinds of fine-grained tailings sand samples at various head pressures are performed separately. The layer-by-layer porosity statistics of the three fine-grained samples are shown in Figure 12.

The following conclusions can be drawn from Figure 12:

(1) With an increasing head pressure of the 30% fine-grained samples, the porosity of the samples in the same area shows an increasing trend from layer to layer. An increase in the seepage force affects the porosity of the tailings sample at each layer. The effect of the rate is obvious. With an increase in the number of slice layers in the z-axis direction, that is, an increase in the number of 2D slice layers along the seepage direction, the tailings sand samples at all the levels of the water head show an increasing trend in the porosity of each layer.
(2) The 50% fine content tailings sand sample gradually increases with head pressure, and the average porosity gradually increases. Compared with the 30% fine-grained tailings sand sample, the porosity of each layer is smaller, indicating that the increase in the fine particle content has a significant effect on the porosity of each layer. It can be seen from the figure that the porosity of the tailings sand sample gradually increases from 200 to 400 layers, while the porosity of the 400 to 600 layers does not change much. This finding indicates that when the water level is between 1 and 2, the sample connected air does not develop, the fine particles do not migrate significantly in the 400–600 layer, and the head porosity of the 200–400 layer of the sample decreases sharply when the head pressure increases to a level 3 head, while the 400–600 layer decreases layer by layer. The porosity increases sharply, indicating that the sample suffered fluid damage.
(3) For the 70% fine content tailings sand sample, the head pressure and average porosity gradually increase. It can be seen from the layer-by-layer porosity curve under the level 2 head pressure that the porosity suddenly increases and then decreases in the areas at approximately 100 and 500 layers, indicating that the migration and accumulation of fine particles in this area are most obvious. When the head pressure is increased to level 3, the layer-by-layer porosity of the tailings sand sample has a sharp increase compared to the layer-by-layer porosity at the level 2 head pressure. Regarding the fluid soil penetration damage, the peak-to-layer porosity appeared in the 300–600-layer area, indicating that the tailings sand sample is the most damaged in this area.

### 3.3. Microstructure Distribution Characteristics of the Particle Size and the Particle Size during Percolation Failure of the Tailings Sand

**Figure 13:**
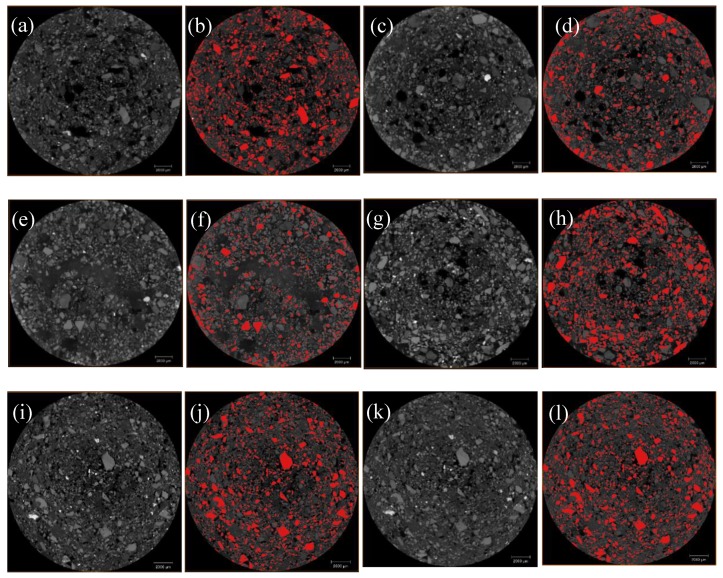

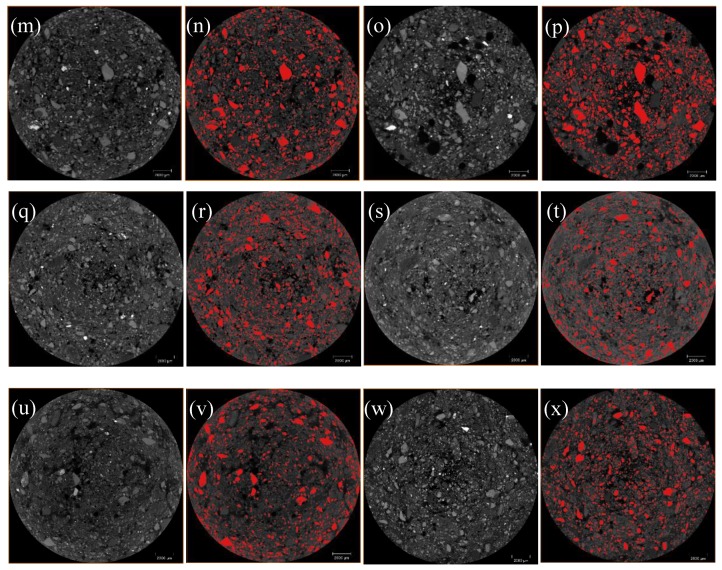
Threshold segmentation of the tailings sand particles. (**a**,**b**) First-level head of the 30% fine content; (**c**,**d**) Second-level head of the 30% fine content; (**e**,**f**) Third-level head of the 30% fine content; (**g**,**h**) Fourth-level head of the 30% fine content; (**i**,**j**) First-level head of the 50% fine content; (**k**,**l**) Second-level head of the 50% fine content; (**m**,**n**) Third-level head of the 50% fine content; (**o**,**p**) Fourth-level head of the 50% fine content; (**q**,**r**) First-level head of the 70% fine content; (**s**,**t**) Second-level head of the 70% fine content; (**u**,**v**) Third-level head of the 70% fine content; (**w**,**x**) Fourth-level head of the 70% fine content.

Based on the Avizo 3D visualization software, the data obtained from CT scans of the three fine-grained tailings sand samples were binarized after threshold segmentation, and tailings sand particles with the highest gray values were extracted, as shown in Figure 13.

Based on the data of tailings sand particles obtained after threshold segmentation, 200 layers of 2D slices were used as a region, and a total of 1000 layers were divided into five regions through the Extract Subvolume command in the 3D visualization software of Avizo. Particle size distributions of 10–20 μm, 20–40 μm, and 40–75 μm were counted.

#### 3.3.1. Thirty Percent Fine Content Sample

**Figure 14:**
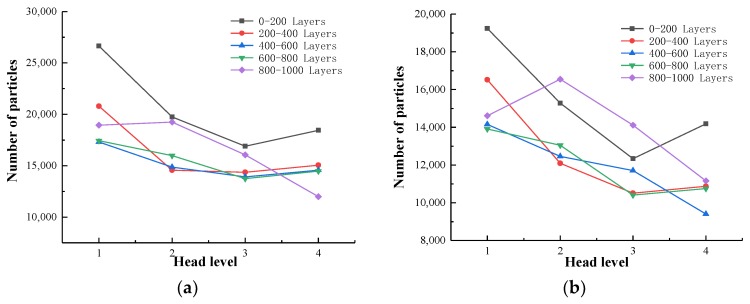

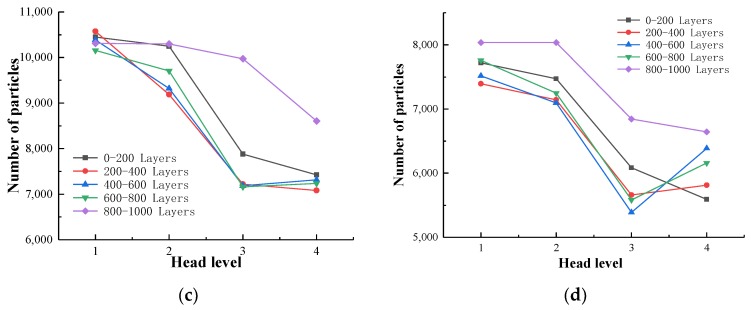
Variation in the number of fine particles percolated with the 30% fine content. (**a**) Particle size: 0–10 µm; (**b**) Particle size: 10–20 µm; (**c**) Particle size: 20–40 µm; (**d**) Particle size: 40–75 µm.

The statistical results are shown in Table 7, Table 8, Table 9 and Table 10 and Figure 14.

From the above chart, the following can be observed:

(1) As the water pressure level increases, the tailings particles with particle sizes of 0 to 75 μm in the same area generally show a gradual decrease in the direction of seepage. The ground moves in the direction of seepage. In the first three levels of the head difference, as the head difference increases, the particle migration becomes more obvious, and the particle size of the fine-grained tailings continues to increase. There is a proportional relationship between the two.
(2) In the first water pressure level to the second water pressure level, as the infiltration damage starts to occur, the tailings particles with particle sizes of 0–20 μm in the same area decrease significantly, and the tailings particles with particle sizes of 20–75 μm decline very slowly. The tailings particles with particle sizes of 0 to 20 μm are most sensitive to the seepage forces during the initial stage of infiltration and destruction. The seepage channels formed during the initial stage of infiltration and destruction are relatively small but can form good flow migration in the seepage channel. However, the tailings with particle sizes of 20 to 75 μm are larger, and thus, the flow is hindered in the pore channel. Additionally, in the 0–400 layers at the bottom of the sample, the particles migrate. This phenomenon is significant, and the downward trend of particles above 400 layers is obviously weakened. The analysis shows that some fine particles at the bottom of the sample migrate to fill the pore space above the 400 layers, and because the head difference is small, the particles stay in the space, resulting in more than 400 layers of the sample. Furthermore, the content of fine particles in the space of 0–20 μm increases, which is an upward trend compared with the 200–400 layers.
(3) From the second water pressure level to the third water pressure level, the 0 to 10 μm particle size tailings particles in the 0–800-layer sample have a slower downward trend, and the 20 to 75 μm size tailings particles begin to rapidly decline. It can be seen that during the infiltration failure process, the seepage channels and pores continue to develop and expand to make the tailings particles with particle sizes of 20 to 75 μm fully flow. In the 800–1000-layer sample, the fine particle content is from 20–75 μm. The upper-level head has already migrated, and thus, the number of particles does not decrease significantly. In contrast, the migration of fine-grained tailings with particle sizes of 40–75 μm is more obvious than that of the 20–40 μm particles, which confirms that the fine-grained tailings sand has migrated. The particle size migration is proportional to the head difference.
(4) From the third water pressure level to the fourth water pressure level, the tailings particles with particle sizes from 0 to 75 μm generally show an increasing trend. The seepage channels and pores of the tailings sample are completely destroyed. After pressure relief, the CT scans show the obvious tailings particle sedimentation in the seepage channel, indicating that a penetrating infiltration failure channel is formed inside.

#### 3.3.2. Fifty Percent Fine Content Sample

**Figure 15:**
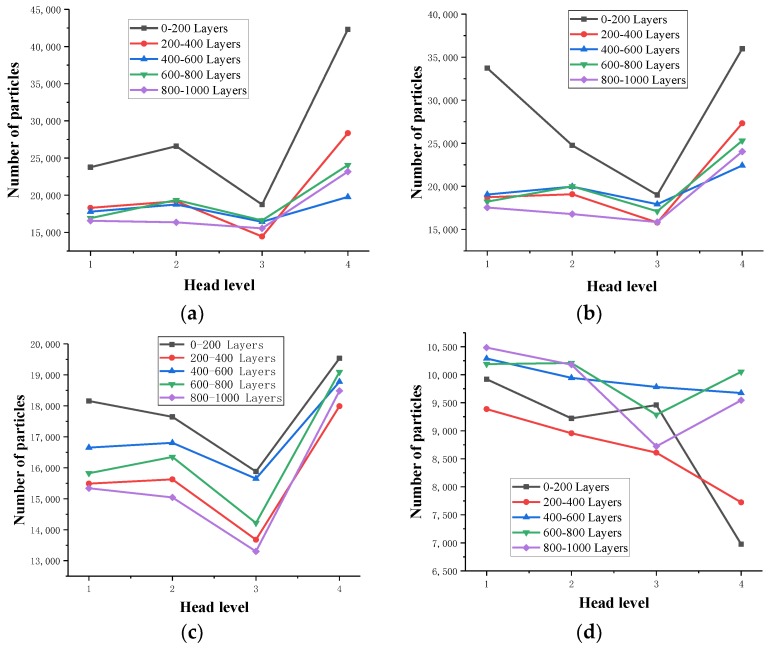
Variation in the number of fine particles percolated with the 50% fine content. (**a**) Particle size: 0–10 µm; (**b**) Particle size: 10–20 µm; (**c**) Particle size: 20–40 µm; (**d**) Particle size: 40–75 µm.

The statistical results of the 50% fine content are shown in Table 11, Table 12, Table 13 and Table 14 and Figure 15.

From the above chart, the following can be observed:

(1) In the first water pressure level to the second water pressure level, with increasing seepage pressure, the tailings particles with particle sizes from 0 to 10 μm in the entire sample begin to increase, indicating that there is 0 in the sample. The connected pore structure of 0–10 µm demonstrates a significant particle migration phenomenon. The fine particle content of 10–20 µm rapidly decreases at the bottom of the scanned sample, and the number increases in areas that are more than 200 layers larger than the scanned area, indicating that the internal pore size of the sample is greater than 10 µm. The pores are not developed, and the particles remain in the pores after migration within this size range, resulting in an increase in the fine particle content of pores of 10–20 µm. Fine particles larger than 20 µm show a certain downward trend as a whole. However, the downward trend is not obvious and almost remains the same, indicating that the connected pores larger than 20 µm in diameter do not develop inside the sample, and there are fewer internal seepage channels.
(2) From the second water pressure level to the third water pressure level, the permeability increases, the internally connected pores of the sample begin to develop, the fine particles of each particle size level begin to migrate and flow, and the overall content decreases.
(3) At the third water pressure level to the fourth water pressure level, the sample is damaged, and the internal pores are fully developed. As the pressure is released, the fine particles at each particle size level in the sample rapidly increase.

#### 3.3.3. Seventy Percent Fine Content Sample

**Figure 16:**
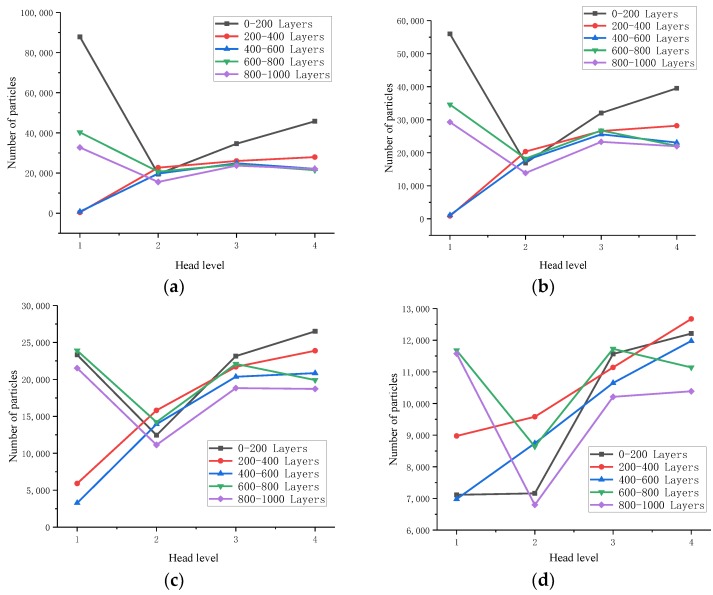
Variation in the number of the fine particles percolated with the 70% fine content. (**a**) Particle size: 0–10 µm; (**b**) Particle size: 10–20 µm; (**c**) Particle size: 20–40 µm; (**d**) Particle size: 40–75 µm.

The statistical results of the 70% fine content are shown in Table 15, Table 16, Table 17 and Table 18 and Figure 16.

(1) In the first water pressure level to the second water pressure level, with increasing seepage pressure, the particle sizes of 0-10 μm, 10–20 µm, and 20–40 µm are exhibited within the range of 0–200 layers. The tailings particles decrease rapidly, while the fine content in the 200–600-layer increases, indicating that the fine content at the bottom migrated to the middle of the sample and that the internal pores of the sample are not well developed during the initial stage of percolation. No continuous percolation channel is formed.
(2) Under the second water pressure level to the third water pressure level, the seepage pressure increases, the migration force between particles increases, the overall content of the fine particles of each particle size increases, the internal pressure increases, and the connected pores begin to develop slowly. The sample will soon be damaged by liquid soil.

#### 3.3.4. Changes in the Total Value of the Fine Content of the Three Samples

**Figure 17:**
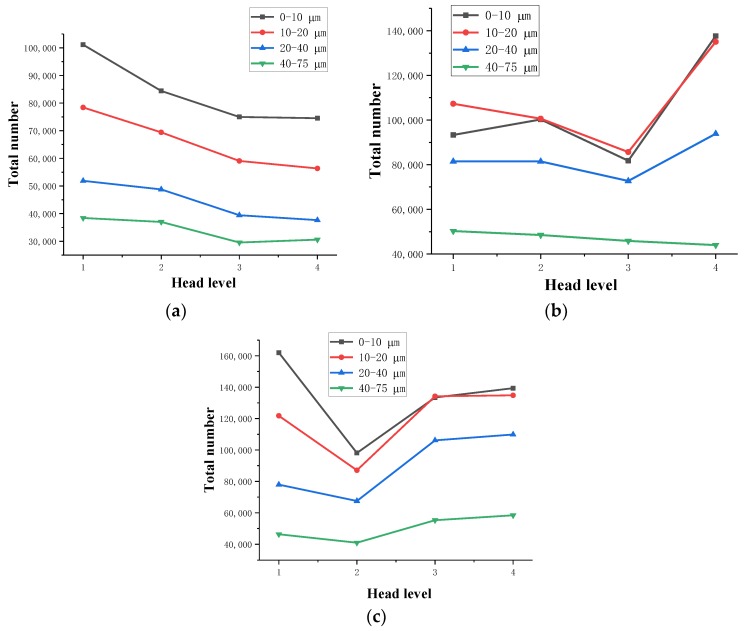
The number of fine particles with different particle sizes. (**a**) 30% fine content; (**b**) 50% fine content; (**c**) 70% fine content.

The statistics regarding the changes in the fine contents in three kinds of fine-grained tailings sand samples during percolation are shown in Table 19, Table 20, Table 21 and Table 22 and Figure 17.

(1) It can be seen that the total content of all fine particles in the 30% fine particle sample decreases during percolation, and the fine particles flow out during percolation. Clearly, a piping-type infiltration failure has occurred.
(2) Regarding the 50% and 70% fine particle contents, all of the fine particle contents of the samples decreased first and then increased rapidly, and the 70% fine particle content sample changes significantly more than the 50% fine particle content sample. At the beginning of the percolation, the fine particles continuously flow out in the seepage direction. As the seepage pressure increases, the fine particles do not have enough pores to flow, and the soil breaks down. After settlement, the fine particle content increases significantly.
(3) Through a comparative analysis, it can be observed that during the percolation of the sample, fine particles migrate along the percolation direction, and the smaller the particles are, the more obvious the migration. The difference is that the number of fine particles will affect the type of osmotic failure of the tailings sand sample. The tailings sand with a fine content near 30% will undergo piping-type osmotic failure, and its fine particle content will continue to decrease. At the 30% fine content, the tailings sand will undergo osmotic failure, its fine particle content will decrease, and then all of the particles will flow.

**Figure 18:**
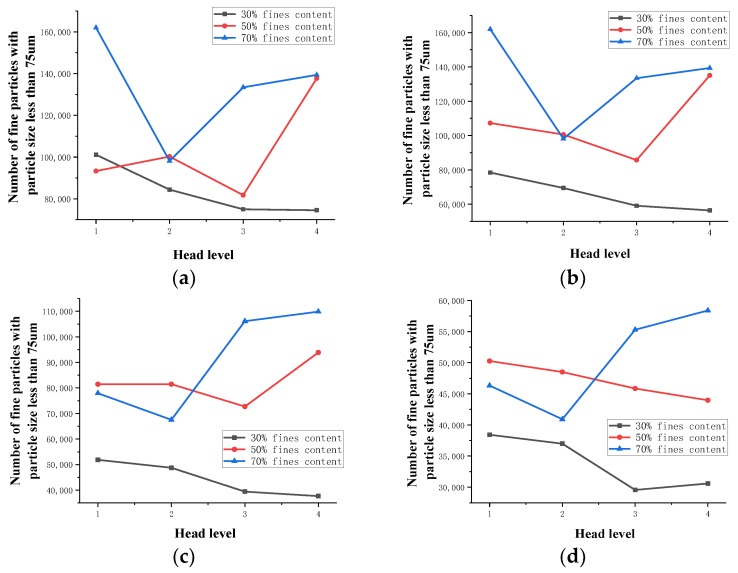
Variation in the particle number during infiltration with different fine contents. (**a**) Particle size: 0–10 µm; (**b**) Particle size: 10–20 µm; (**c**) Particle size: 20–40 µm; (**d**) Particle size: 40–75 µm.

Comparing the fine particle content migration with the different fine particle contents and different particle diameters, it can be seen that the occurrence of fine particle tailings sand permeation failure is closely related to the fine particle content migration and the formation of the seepage channels. The relationship between the changes in the fine particle content of different particle sizes and the head pressure during the seepage process of tailing sand samples with different fine particle contents is shown in Figure 18.

From Figure 18, the following can be observed:

(1) The content of the 30% fine particles shows a downward trend, which indicates that the internal seepage channels and connected pore volumes are constantly expanding, and the tailings sand samples undergo piping-type infiltration failure.
(2) The content of 50% of fine particles shows a decreasing trend at the beginning of the seepage, but the overall change is not large. With increasing water pressure, the sample suffers soil damage, the internal seepage channels are not obvious, and the connected pore volume slowly increases.
(3) For the 70% fine particle content, during the early stage of seepage, due to a large number of fine particles, the 0–20 µm fine particle content rapidly decreases, but the internally connected pore volume grows less. As the seepage pressure increases, the flow-type soil infiltration is quickly damaged.

**Figure 19:**
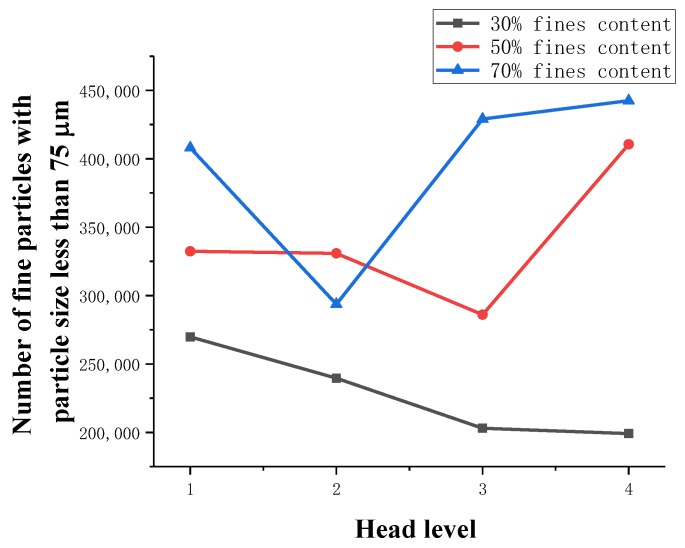
Fine particle content as a function of the number of infiltration processes.

During the seepage process, the changes in the number of fine-grained particles under the heads at all levels are shown in Table 23 and Figure 19.

From the above chart, the following can be observed:

(1) For the 30% fine particle content, the fine particle content of the sample decreases continuously at all levels of the seepage pressure until it stabilizes, and piping damage occurs.
(2) For the 50% fine particle content, the total number of fine particles of the sample slowly decreases at the beginning of the seepage. As the seepage pressure increases, the rate of fine particles decreases gradually. After the infiltration failure of fluid soil occurs, the fine particle content rapidly increases due to settlement.
(3) For the 70% fine particle content of the sample at the beginning of the seepage, due to a large number of fine particles, the total number of fine particles decreases rapidly. After infiltration and destruction of the raw fluid soil, the fine particle content increases rapidly due to sedimentation.
(4) Through comparative analysis, it can be found that for samples with a small number of fine particles, since the connected pores are relatively developed, there are continuous fine particle seepage channels, and the fine particle content is also small. During the seepage process, fine particles will continue to migrate. The type of osmotic failure caused by effluent flow is a piping failure. For samples with a large number of fine particles, the internal communication pores are poorly developed, there is no continuous percolation channel, and the sample has a large number of fine particles. With increasing water pressure, cracks will appear in the sample, the whole flow will flow in the direction of seepage, and the soil will be damaged.

**Figure 20:**
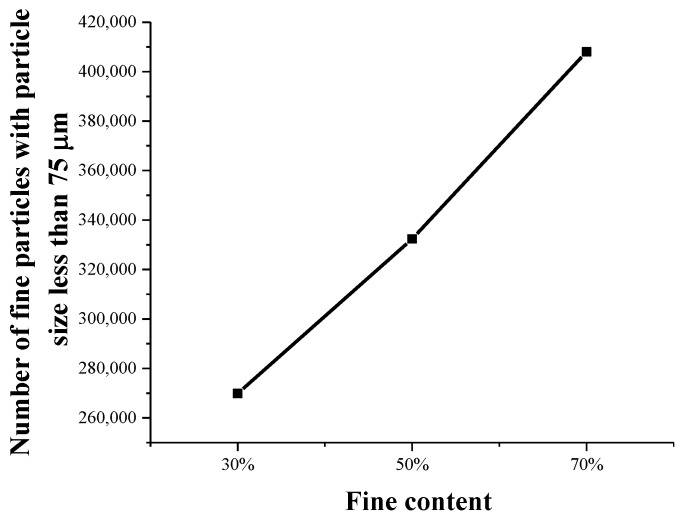
Relationship between the proportion of the fine content and the initial content of fine particles.

**Figure 21:**
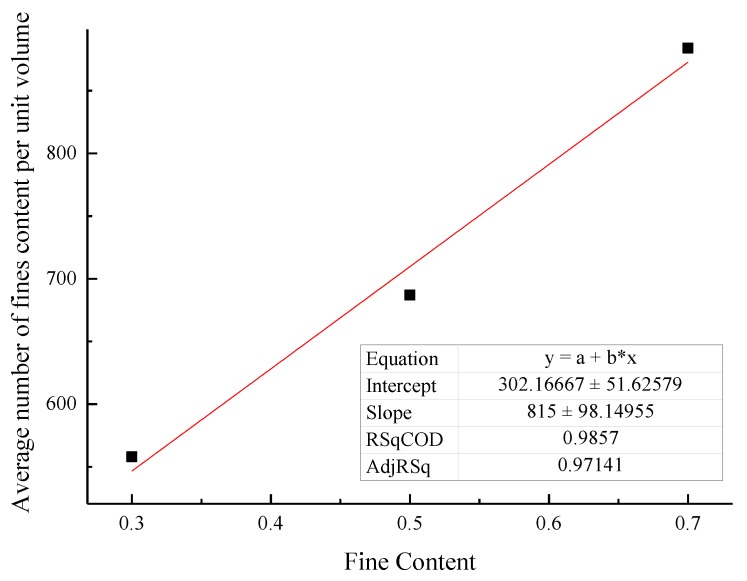
Relationship between the proportion of the fine content and the average content of fine particles per unit volume.

Both the relationship between different proportions of fine-grained tailings sand samples and the initial number of fine particles and the relationship between different proportions of fine-grained tailings sand samples and the average number of fine-grained contents per unit volume are analyzed, as shown in Figure 20 and Figure 21.

It can be seen that there is a clear linear relationship between the content of fine particles and the total number of fine particles. At the same time, the analysis of the fine particle content in the unit volume is the ratio of the fine particle content of the sample to the sample volume. Through data fitting, it can be found that the percentage of fine particle content and the fine particle content in the unit volume exhibit a very obvious linear correlation. Therefore, the fine particle content per unit volume can be used as the mesoparameter of samples with different fine particle contents.

### 3.4. Macroscopic and Mesofactor Analysis of the Seepage Process

**Figure 22:**
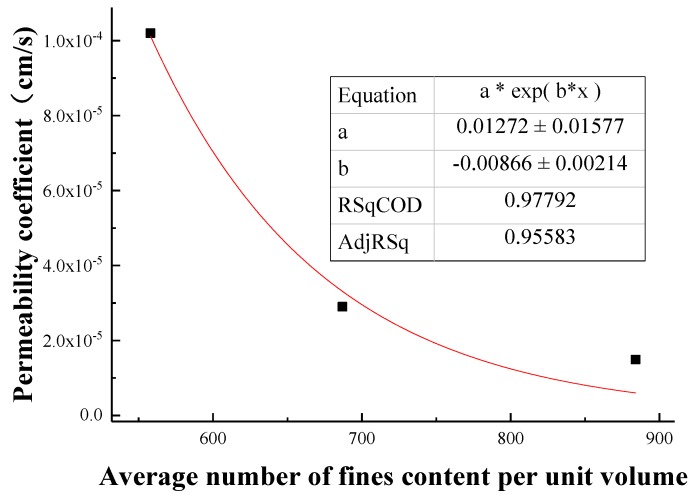
Fitting curve of the fine particle content and permeability coefficient per unit volume.

**Figure 23:**
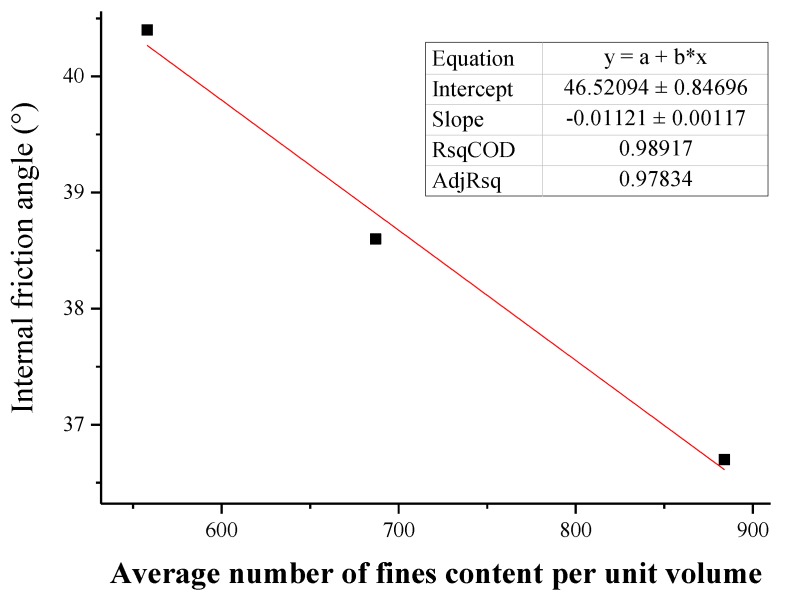
Fitting curve of the fine particle content and internal friction angle per unit volume.

**Figure 24:**
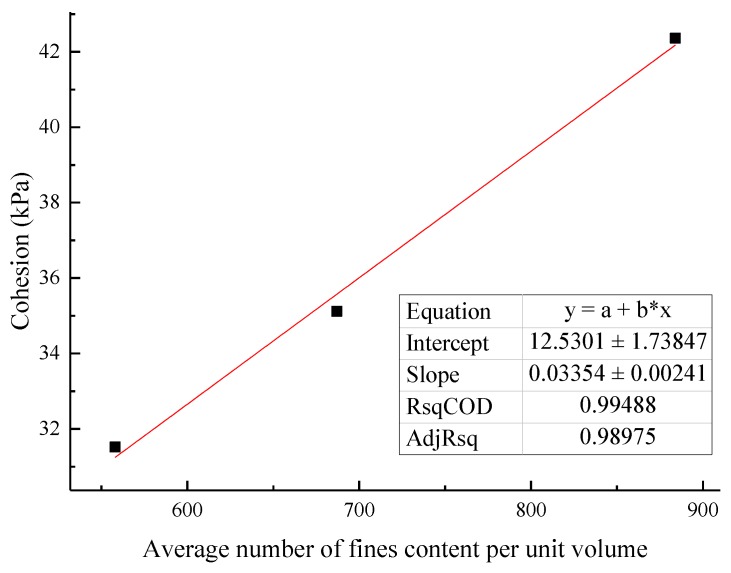
Fitting curve of the fine particle content and cohesion per unit volume.

**Figure 25:**
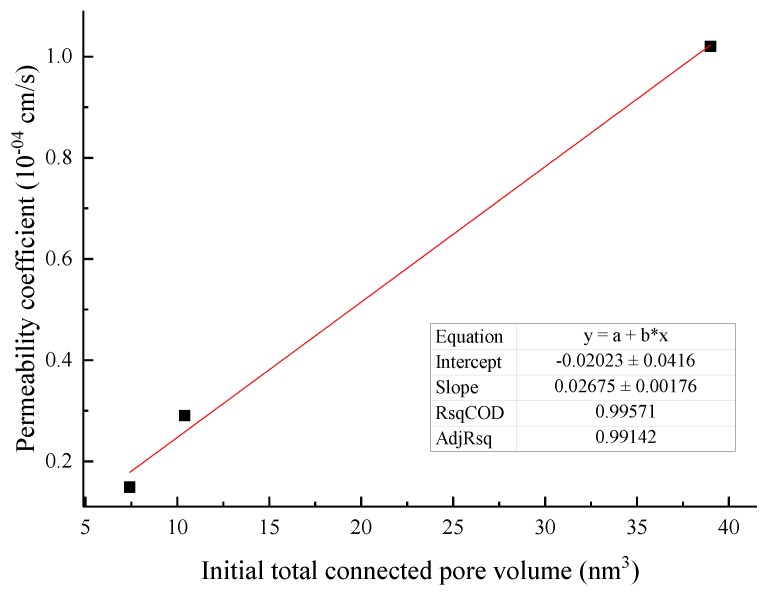
Fitting curve of the connected pore volume and permeability coefficient.

**Figure 26:**
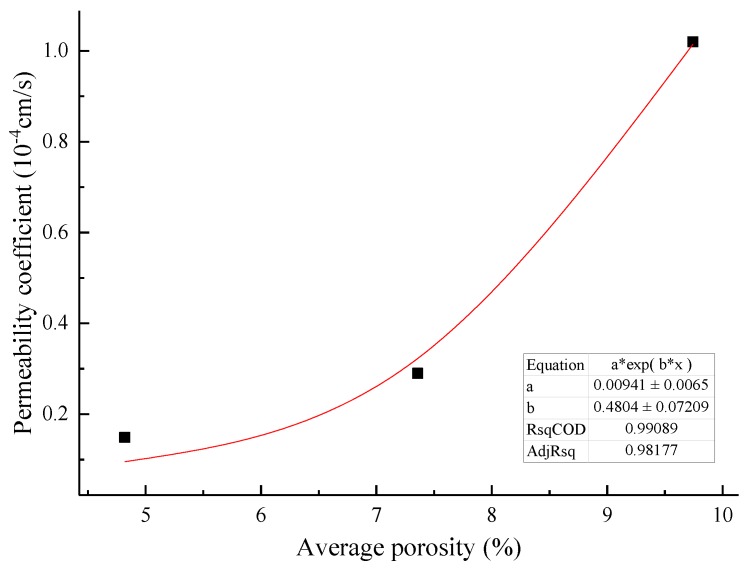
The fitting curve of the average porosity and permeability coefficient.

Based on the macrophysical and mechanical properties, a comparative analysis of the parameters of the microstructure characterization of the tailings sand sample is performed. The macro- and mesofactor comparison table is shown in Table 24. The relationship between the fitted macro- and mesofactors is shown in Figure 22, Figure 23, Figure 24, Figure 25 and Figure 26.

The following conclusions can be drawn from the above chart:

(1) Analyzing the data relationship between the content of fine particles per unit volume and the permeability coefficient, internal friction angle, and cohesion that affect the seepage and failure of the tailings sand sample macroscopically, the following data characteristics can be found:

With increasing fine particle content per unit volume, the permeability coefficient and the internal friction angle gradually decrease. The former shows a more obvious exponential function relationship, while the latter shows a more obvious positive correlation with a linear function. For the fitting formula, see Equations (1) and (2).

(1) $$k=a_{1}\times e^{b_{1}n}$$

where *k* is the internal friction angle, cm·s^−1^; *n* is the fine particle content per unit volume; *a*_1_ is a material parameter, which can be obtained by fitting the experimental data; and *b*_1_ is a material parameter, which can be obtained by fitting the experimental data.

(2) $$\;\varphi =a_{2}{+b}_{2}\times n$$

where *φ* is the permeability coefficient, °; *n* is the connected pore volume, μm^3^; *a*_2_ is a material parameter, which can be obtained by fitting the experimental data; and *b*_2_ is a material parameter, which can be obtained by fitting the experimental data.

As the content of fine particles in a unit volume increases, the cohesion gradually increases, and the two show a relationship in the form of an exponential function. The fitting formula is shown in Equation (3):

(3) $$c=a\times e^{bn}$$

where *c* is the cohesion, kPa; *n* is the fine particle content per unit volume; *a* is a material parameter, which can be obtained by fitting the experimental data; and *b* is a material parameter, which can be obtained by fitting the experimental data.

(2) The connected pore volume and the average porosity of the sample can be used as the macro- and micropore structure characteristics of the sample. The data relationship analysis reveals the following data characteristics:

The initial volume of connected pores has a positive linear correlation with the permeability coefficient. The fitting formula is shown in Equation (4).

(4) k=a+b×V

where *k* is the permeability coefficient, cm·s^−1^; *V* is the connected pore volume, μm^3^; *a* is a material parameter, which can be obtained by fitting the experimental data; and *b* is a material parameter, which can be obtained by fitting the experimental data.

The fitting curve of the average porosity and permeability coefficient shows an exponential relationship, and it’s fitting formula is shown in Equation (5).

(5) $$k=a_{1}\times n^{b_{1}}$$

where *k* is the permeability coefficient, cm·s^−1^; *n* is the average porosity of the sample, %; *a*_1_ is a material parameter, which can be obtained by fitting the experimental data; and *b*_1_ is a material parameter, which can be obtained by fitting the experimental data.

Through the analysis of the above data, it can be found that the number of fine particles in a unit volume and the connected pore volume can be used as microstructure parameters to establish a relationship with the macroscopic physical and mechanical properties of the sample. The observation angle is combined with the macrophenomena for comparative analysis.

## 4. Discussion

### 4.1. Piping-Type Infiltration Failure

Piping is a very complicated failure process. Some scholars believe that the soil mainly consists of two parts; one is the skeleton of the soil, and the other is the movable fine particles contained in the soil skeleton. Therefore, under the action of water flow, through the pores in the soil skeleton, fine particles move with the action of water flow [18].

When the drag force of the water flow in the soil [19,20,21,22] breaks through the fine static balance between the soil particles, the effective stress between the skeletons decreases and the loss of fine particles occurs inside the soil, forming a large number of infiltration quicksand channels. The rearrangement and deposition of soil particles lead to changes in the microstructure and mechanical properties of the soil [23], and the stress redistribution between the framework particles further reduces the overall stability of the soil, causing piping failure. The development of piping is a process that gradually erodes from downstream particle loss to upstream particle loss [24]. Some scholars have also proposed a capillary model for piping in non-cohesive soils, suggesting that the permeability during piping is significantly affected by fine particle loss [25].

Summarizing the microscopic structural characteristics of the piping-type infiltration failure sample during the seepage process, it can be found that the total amount of fine particles in the sample shows a downward trend, which explains the phenomenon of the continuous loss of fine particles. Its layer-by-layer porosity also increases uniformly with the flow of seepage, and the volume of connected pores also increases, but the number of pores decreases, indicating that small micropores are connected to large pores due to the loss of fine particles. The skeleton structure of the sample was changed, which led to changes in the mechanical and permeability characteristics.

### 4.2. Soil-Type Infiltration Failure

The mechanism of soil-type infiltration failure can be analyzed with the dominant flow produced by the seepage in the sample [26]. Dominant flow refers to the concentrated and rapid flow of water in the pores in a few areas, while the velocity in other areas is much smaller than the velocity in the fast channel [27]. The formation and development of the dominant flow have spatiotemporal variability [28,29].

In this test, the spatial variability of the dominant flow in the sample created a cyclic process of “formation → development → disappearance → reformation” of the dominant flow. At the beginning of the seepage test, the seepage force is small, the dominant flow is not obvious, and fine particles cannot be dragged to migrate. As the head pressure is gradually increased, the seepage force begins to increase, the fine particles begin to redistribute under the drag of the seepage force, and the permeability coefficient of the sample begins to increase slowly. The randomness of the direction of the pore flow velocity is represented by the non-uniformity of permeation in the macrostructure. Under the non-uniform percolation, large local pores and weak links appear in the sample, and thus, the dominant flow is generated. The generation of the dominant flow provides a channel for the movement of fine particles, and the fine particles gradually move towards the upper part of the channel under the drag of the current. The fine particles in the soil around the dominant flow channel are lost, and the structure is destroyed. The coarse particles will collapse, thus blocking the dominant flow channel and forming an anti-filtration layer, and the dominant flow channel will disappear. The permeability coefficient of the reverse filtration layer is small, and the permeation flow rate is also low. There will be the continuous accumulation of particles, which makes the permeability coefficient of the reverse filtration layer smaller and smaller, and the osmotic pressure on both sides of the reverse filtration layer gradually increases. When the osmotic pressure reaches a critical value, a new dominant flow channel is formed. This explains the abrupt change in the layer-by-layer porosity of the mesostructure that seeps through; that is, the formation of the dominant flow causes the fine particles to migrate to the upper part of the channel and the layer-by-layer porosity increases. Coarse particles collapse, the dominant flow disappears, fine particles begin to accumulate, and the porosity decreases continuously from layer to layer. At the same time, the phenomenon that the coarse particle size at the bottom of the sample increases and the fine particle size decreases sharply when the soil failure occurs in the sample is also explained.

At the beginning of the formation of the dominant flow, the overall permeability coefficient of the soil does not change much, and the space for expansion of the dominant flow is limited. Therefore, the dominant flow can develop and transfer only in local areas. The formation and development of the dominant flow are accompanied by the seepage deformation and seepage failure of the soil body. The dominant flow will continue to find weaker areas in the soil body to form new dominant flows. In the process of slowly increasing the permeability coefficient, new and larger dominant streams continue to emerge. In the cycle of continuous formation → development → disappearance → reformation of the dominant flow, the soil seepage deformation gradually deepens, the soil structure is gradually destroyed, and the macroscopic infiltration velocity in the soil gradually increases. When the critical hydraulic gradient of the soil is reached, at the same time, the soil body was damaged by liquid soil. The structure of the sample has been severely disturbed after the failure of fluid soil occurred, and more microcracks have been formed in the sample, making it easier to move the particles in the soil. The content of fine particles increases sharply, and the number of pores also increases dramatically.

## 5. Conclusions

(1) There are significant differences in the microscopic structural characterization between piping-type infiltration failure and fluid soil-type infiltration failure. The 30% fine-grained tailings sand sample has a type of piping failure, and the porosity gradually increases from layer to layer during the seepage process. Small pores of 0 to 10 μm are easily penetrated into pores larger than 10 μm. The total pores, both the number and the number of fine particles, showed a decreasing trend. The infiltration failure types for between 50% and 70% of the fine-grained tailings sand samples were fluid soil-type, and the porosity layer by layer in the middle of the sample significantly increased during the seepage process. The total pore number increased first and then decreased, while the fine particle content decreased first and then increased sharply.
(2) The piping-type infiltration failure and connected pore development are significantly stronger than the fluid soil-type infiltration failure. The evolution characteristics of the connected pores of the piping-type osmotic failure and the fluid-type osmotic failure are basically similar; that is, the connected pore structure develops from the sample center to the surroundings.
(3) It is found through experiments that there is a certain functional relationship between the microstructure index of tailings sand, micromechanics, and the permeability index. The fine particle content per unit volume demonstrates a relationship in the form of an exponential function with the permeability coefficient and cohesion. The fine particle content per unit volume has a linear negative relationship with the internal friction angle. The initial total connected pore volume has a positive linear correlation with the permeability coefficient. The average porosity has an exponential relationship with the permeability coefficient. Combining the relationship between macrophysical and mechanical parameters and microstructure parameters, a connection has been established for the comparative analysis of macrophenomena from the microstructure to the infiltration and destruction of tailings sand in the future.

## Figures and Tables

**Table 1:** CT scan parameters.

Sample Name	Test Voltage/kV	Test Current/μA	Exposure Time/s	Resolution/μm	Pixel	X-ray Source to Sample Distance (sod)/mm	Distance from X-ray Source to Flat Panel Detector (sdd)/mm
Tailings sand sample	140	70	0.42	6.16	1800 × 1800 × 1536	13.6	279.6

**Table 2:** Head loading values at various levels.

Head Level	Loading Head Level/cm		
	30% Fine Content	50% Fine Content	70% Fine Content
1	29.0	32.0	48.0
2	37.0	43.0	52.0
3	39.0	46.0	56.0
4	42.0	51.0	58.0

**Table 3:** Seepage velocity and permeability coefficient during the test.

Fine Content	Head Level	Flow Rate cm^3^/s	Permeability Coefficient cm/s
30%	1	8.34 × 10^−3^	9.95 × 10^−4^
	2	1.23 × 10^−2^	1.14 × 10^−3^
	3	2.78 × 10^−2^	2.45 × 10^−3^
	4	8.34 × 10^−2^	6.52 × 10^−3^
50%	1	7.59 × 10^−3^	8.18 × 10^−4^
	2	1.30 × 10^−2^	1.04 × 10^−3^
	3	2.98 × 10^−2^	2.23 × 10^−3^
	4	5.96 × 10^−2^	4.01 × 10^−3^
70%	1	6.95 × 10^−3^	6.30 × 10^−4^
	2	1.30 × 10^−2^	8.60 × 10^−4^
	3	1.67 × 10^−2^	1.02 × 10^−3^
	4	6.95 × 10^−2^	3.84 × 10^−3^

**Table 4:** Statistics of the number of pores in the seepage process with the 30% fine content.

Head Level	Pore Size D/µm					Total Number of Pores	Total Connected Pore Volume (µm^3^)
	0–10	10–20	20–40	40–80	>80		
1	210,450	129,578	35,057	9092	4557	388,734	3.9 × 10^10^
2	166,833	138,197	40,293	9347	3983	358,653	6.05 × 10^10^
3	133,557	156,602	41,543	10,553	4702	346,957	6.5 × 10^10^
4	81,410	140,923	44,661	24,151	6170	297,315	8.34 × 10^10^

**Table 5:** Statistics of the number of pores in the seepage process with the 50% fine content.

Head Level	Pore Size D/µm					Total Number of Pores	Total Connected Pore Volume (µm^3^)
	0–10	10–20	20–40	40–80	>80		
1	126,404	87,362	44,256	23,020	9373	290,415	1.04 × 10^10^
2	151,873	103,059	31,162	22,938	8224	317,256	2.32 × 10^10^
3	142,467	97,344	41,445	19,447	6450	307,153	2.75 × 10^10^
4	283,942	184,156	55,797	18,723	5370	547,988	3.47 × 10^10^

**Table 6:** Statistics of the number of pores in the seepage process with the 70% fine content.

Head Level	Pore Size D/µm					Total Number of Pores	Total Connected Pore Volume (µm^3^)
	0–10	10–20	20–40	40–80	>80		
1	144,629	92,459	51,268	27,397	8370	324,123	7.41 × 10^9^
2	121,473	79,449	28,819	12,602	3745	246,088	1.43 × 10^10^
3	239,642	169,238	62,833	20,485	2605	494,803	4.8 × 10^10^
4	555,965	321,095	83,504	19,136	2809	982,509	5.74 × 10^10^

**Table 7:** Variation in the number of fine particles percolated with the 30% fine content (0–10 µm).

Head Level	Layers					Total Number of Particles
	0–200 Layers	200–400 Layers	400–600 Layers	600–800 Layers	800–1000 Layers	0–10 μm
1	26,653	20,791	17,301	17,441	18,944	101,130
2	19,751	14,573	14,862	15,978	19,242	84,406
3	16,894	14,363	13,917	13,750	16,062	74,986
4	18,444	15,048	14,558	14,469	12,000	74,519

**Table 8:** Variation in the number of fine particles percolated with the 30% fine content (10–20 µm).

Head Level	Layers					Total Number of Particles
	0–200 Layers	200–400 Layers	400–600 Layers	600–800 Layers	800–1000 Layers	10–20 μm
1	19,241	16,522	14,149	13,905	14,608	78,425
2	15,282	12,091	12,460	13,044	16,546	69,423
3	12,337	10,511	11,703	10,406	14,108	59,065
4	14,183	10,871	9400	10,753	11,156	56,363

**Table 9:** Variation in the number of fine particles percolated with the 30% fine content (20–40 µm).

Head Level	Layers					Total Number of Particles
	0–200 Layers	200–400 Layers	400–600 Layers	600–800 Layers	800–1000 Layers	20–40 μm
1	10,450	10,572	10,384	10,157	10,312	51,875
2	10,248	9187	9322	9704	10,299	48,760
3	7881	7220	7186	7160	9973	39,420
4	7423	7080	7315	7237	8605	37,660

**Table 10:** Variation in the number of fine particles percolated with the 30% fine content (40–75 µm).

Head Level	Layers					Total Number of Particles
	0–200 Layers	200–400 Layers	400–600 Layers	600–800 Layers	800–1000 Layers	40–75 μm
1	7721	7394	7518	7758	8038	38,429
2	7472	7144	7095	7247	8036	36,994
3	6086	5661	5388	5582	6844	29,561
4	5591	5814	6387	6161	6644	30,597

**Table 11:** Variation in the number of fine particles percolated with the 50% fine particle content (0–10 µm).

Head Level	Layers					Total Number of Particles
	0–200 Layers	200–400 Layers	400–600 Layers	600–800 Layers	800–1000 Layers	0–10 µm
1	23,766	18,299	17,785	16,910	16,568	93,328
2	26,602	19,188	18,753	19,365	16,355	100,263
3	18,741	14,446	16,426	16,607	15,548	81,768
4	42,318	28,355	19,772	24,064	23,177	137,686

**Table 12:** Variation in the number of fine particles percolated with the 50% fine particle content (10–20 µm).

Head Level	Layers					Total Number of Particles
	0–200 Layers	200–400 Layers	400–600 Layers	600–800 Layers	800–1000 Layers	10–20 µm
1	33,729	18,744	19,050	18,225	17,541	107,289
2	24,766	19,084	19,974	19,998	16,777	100,599
3	18,999	15,798	17,927	17,105	15,843	85,672
4	35,974	27,324	22,419	25,302	24,038	135,057

**Table 13:** Variation in the number of fine particles percolated with the 50% fine particle content (20–40 µm).

Head Level	Layers					Total Number of Particles
	0–200 Layers	200–400 Layers	400–600 Layers	600–800 Layers	800–1000 Layers	20–40 μm
1	18,156	15,488	16,652	15,824	15,339	81,459
2	17,645	15,627	16,806	16,350	15,042	81,470
3	15,878	13,677	15,650	14,216	13,299	72,720
4	19,534	17,989	18,778	19,091	18,482	93,874

**Table 14:** Variation in the number of fine particles percolated with the 50% fine particle content (40–75 µm).

Head Level	Layers					Total Number of Particles
	0–200 Layers	200–400 Layers	400–600 Layers	600–800 Layers	800–1000 Layers	40–75 μm
1	9920	9387	10,290	10,190	10,485	50,272
2	9221	8956	9944	10,209	10,179	48,509
3	9459	8609	9782	9287	8721	45,858
4	6976	7723	9674	10,053	9544	43,970

**Table 15:** Variation in the number of fine particles percolated with the 70% fine particle content (0–10 µm).

Head Level	Layers					Total Number of Particles
	0–200 Layers	200–400 Layers	400–600 Layers	600–800 Layers	800–1000 Layers	0–10 µm
1	87,828	391	766	40,270	32,733	161,988
2	19,479	22,673	19,724	20,699	15,542	98,117
3	34,604	26,022	24,874	24,237	23,683	133,420
4	45,777	27,944	22,124	21,434	22,105	139,384

**Table 16:** Variation in the number of fine particles percolated with the 70% fine particle content (10–20 µm).

Head Level	Layers					Total Number of Particles
	0–200 Layers	200–400 Layers	400–600 Layers	600–800 Layers	800–1000 Layers	10–20 μm
1	55,980	841	1118	34,591	29,263	121,793
2	16,891	20,360	17,716	18,240	13,863	87,070
3	32,021	26,572	25,585	26,746	23,308	134,232
4	39,505	28,175	23,089	22,083	21,968	134,820

**Table 17:** Variation in the number of fine particles percolated with the 70% fine particle content (20-40 µm).

Head Level	Layers					Total Number of Particles
	0–200 Layers	200–400 Layers	400–600 Layers	600–800 Layers	800–1000 Layers	20–40 μm
1	23,315	5911	3267	23,898	21,525	77,916
2	12,441	15,808	13,941	14,219	11,125	67,534
3	23,148	21,702	20,354	22,096	18,821	106,121
4	26,506	23,883	20,851	19,905	18,711	109,856

**Table 18:** Variation in the number of fine particles percolated with the 70% fine particle content (40–75 µm).

Head Level	Layers					Total Number of Particles
	0–200 Layers	200–400 Layers	400–600 Layers	600–800 Layers	800–1000 Layers	40–75 μm
1	7115	8975	6980	11,679	11,576	46,325
2	7160	9582	8743	8640	6796	40,921
3	11,566	11,140	10,648	11,728	10,212	55,294
4	12,209	12,674	11,978	11,141	10,387	58,389

**Table 19:** Fine particle content change during seepage (0–10 µm).

Head Level	30% Fine Content	50% Fine Content	70% Fine Content
1	101,130	93,328	161,988
2	84,406	100,263	98,117
3	74,986	81,768	133,420
4	74,519	137,686	139,384

**Table 20:** Fine particle content change during seepage (10–20 µm).

Head Level	30% Fine Content	50% Fine Content	70% Fine Content
1	78,425	107,289	161,988
2	69,423	100,599	98,117
3	59,065	85,672	133,420
4	56,363	135,057	139,384

**Table 21:** Fine particle content change during seepage (20–40 µm).

Head Level	30% Fine Content	50% Fine Content	70% Fine Content
1	51875	81459	77916
2	48760	81470	67534
3	39420	72720	106121
4	37,660	93,874	109,856

**Table 22:** Fine particle content change during seepage (40–75 µm).

Head Level	30% Fine Content	50% Fine Content	70% Fine Content
1	38,429	50,272	46,325
2	36,994	48,509	40,921
3	29,561	45,858	55,294
4	30,597	43,970	58,389

**Table 23:** Fine particle content change during seepage (0–75 µm).

Head Level	30% Fine Content	50% Fine Content	70% Fine Content
1	269,859	332,347	408,023
2	239,583	330,842	293,643
3	203,031	286,018	429,067
4	199,139	410,587	442,449

**Table 24:** Macro- and mesofactor comparison table.

Fine Content	Fine Content Total Number of Particles	Average Number of Fine Content Per Unit Volume	Average Porosity %	Total Volume of Initial Connected Pores	Permeability Coefficient (cm·s^−1)^	Internal Friction Angle ϕ/°	Cohesion c/kPa
30%	269,859	558	9.74342101	3.90 × 10^10^	1.02 × 10^−4^	40.4	31.52
50%	332,347	687	7.35860292	1.04 × 10^10^	2.90 × 10^−5^	38.6	35.12
70%	408,023	884	4.81893178	7.41 × 10^9^	1.49 × 10^−5^	36.7	42.36
